# Human Cases of Methicillin-Resistant *Staphylococcus aureus* CC398, Finland

**DOI:** 10.3201/eid1610.091571

**Published:** 2010-10

**Authors:** Saara Salmenlinna, Outi Lyytikäinen, Anni Vainio, Anna-Liisa Myllyniemi, Saara Raulo, Mari Kanerva, Merja Rantala, Katariina Thomson, Jaana Seppänen, Jaana Vuopio

**Affiliations:** Author affiliations: National Institute for Health and Welfare, Helsinki, Finland (S. Salmenlinna, O. Lyytikäinen, A. Vainio, J. Vuopio);; Finnish Food Safety Authority, Helsinki (A.-L. Myllyniemi, S. Raulo, J. Seppänen);; Helsinki University Central Hospital, Helsinki (M. Kanerva);; University of Helsinki, Helsinki (M. Rantala, K. Thomson)

**Keywords:** bacteria, antimicrobial resistance, staphylococci, zoonoses, MRSA CC398, limited animal contact, unrecognized transmission chains, Finland, dispatch

## Abstract

Nationwide surveillance identified 10 human isolates of methicillin-resistant *Staphylococcus aureus* clonal complex (CC) 398. Further typing in comparison with animal isolates identified 4 clusters: 1 related to a horse epidemic and 3 to persons who had no direct contact with animals or each other. These findings may indicate unrecognized community transmission.

Animals may serve as a reservoir for methicillin-resistant *Staphylococcus aureus* (MRSA). The MRSA lineage clonal complex (CC) 398 has been reported to be common among pigs (*1**–**3*) and has also been found among other animal species. Occupational exposure to animals has been recognized as a new risk factor for MRSA (*4**,**5*). In Finland, MRSA has occasionally been detected in pets and farm animals. MRSA CC398 was first recognized in Finland in 2007 and involved a veterinary hospital epidemic of 13 horses and 1 employee. Since then, MRSA CC398 has also appeared in other persons and has been found in pig samples. The objective of this study was to recognize possible connections of emerging MRSA CC398.

## The Study

Nationwide surveillance of MRSA in Finland, including notification for human cases and isolate characterization, was started in 1995 (*6*). From 1995 through April 2009, a total of 10,615 nonduplicate human isolates of MRSA were typed by using pulsed-field gel electrophoresis (PFGE); 1 in 2007, 6 in 2008, and 3 in 2009 (n = 10, 0.09%) were nontypable, a typical feature of MRSA CC398 isolates (*7*). Samples of animal origin are usually investigated clinically and through official surveys. No systematic national MRSA surveillance for animals exists, but it is possible to send suspected isolates for confirmation. After the horse epidemic in 2007, some admission screening of animals started at the veterinary hospital where the epidemic occurred. From January 2007 through April 2009, a total of 35 animal MRSA isolates were typed by PFGE; 20 (57%) were nontypable.

For this study, all human and animal isolates of MRSA nontypable by *Sma*I PFGE were further analyzed by testing antimicrobial drug susceptibility against 14 different antimicrobial drug groups, typing the *ccr* and *mec* complex regions within the staphylococcal cassette chromosome *mec* (SCC*mec*) by PCR (*8*), detecting the presence of Panton-Valentine leukocidin (PVL) genes (*9*), *spa* typing, and PFGE by using *Apa*I as the restriction endonuclease. *Apa*I PFGE was performed as described (*10*), except that the restriction digestion was performed with *Apa*I at 30°C for 4 h, and initial and final switching times of 5 s and 15 s, respectively, were used for a 20-h PFGE run. Multilocus sequence typing (MLST) (*11*) was performed on representative isolates of each different combination of origin (human vs. animal species), *spa* type, and SCC*mec*.

For persons whose MRSA isolate was nontypable by *Sma*I PFGE, a structured questionnaire was used to inquire about the presence of commonly known healthcare-related risk factors for MRSA (Technical Appendix). These data were collected from infection control nurses at relevant healthcare districts or directly from patients by telephone interview.

PFGE-nontypable isolates were found in samples from 10 humans, 13 horses, and 7 pigs. Based on combined results from *spa* typing, SCC*mec*, and PVL PCR, 1 single t2922 isolate and 4 clusters of isolates were identified: 14 isolates (t011, SCC*mec*IV) related to the horse epidemic; 3 human isolates (t011, SCC*mec*V) from 2 hospital districts (A and B); 5 human isolates (t034, SCC*mec*V, PVL positive) from 1 hospital district (C); and 7 isolates (t108, SCC*mec*V) from pigs (Table 1). MLST analysis identified sequence type (ST) 398 in all but 1 isolate. This isolate had ST1375, a double locus variant of ST398. In *Apa*I PFGE, 3 groups were identified (Figure). The human t034 isolates from 1 hospital district (C) fell into 1 group. Although all but 1 of the human t011 isolates were clustered together with the pig t108 isolates, subtype-level differences were still detected within this group. Isolates from the horse epidemic formed the third group, showing subtype-level difference from the t2922 human isolate. All isolates were resistant to tetracycline. Resistance patterns to erythromycin, gentamicin, tobramycin, and clindamycin (inducible or noninducible) varied but followed the same grouping as in *Apa*I PFGE (Table 1).

**Table 1:** MRSA CC398 clusters, Finland, January 2007–April 2009*

Cluster	Year	No. isolates	Origin	*spa* type	SCC*mec*	MLST	Antimicrobial drug resistance†	Healthcare district
1	2007	1	Human	t011	IV	398	tet, gen, tob	A
	2007	13	Horse	t011	IV	398	tet, gen, tob	
2	2008	3	Human	t011	V	1375	tet, ery, cli	A, B
Single	2008	1	Human	t2922	V	398	tet, ery, cli	A
3	2008–09	5	Human	t034	V	398	tet, ery, cli (inducible)	C
4	2008–09	7	Pig	t108	V	398	tet, ery, cli	

*MRSA, methicillin-resistant *Staphylococcus aureus*; CC, clonal complex; SCC*mec*, staphylococcal cassette chromosome *mec*; MLST, multilocus sequence typing; tet, tetracycline; gen, gentamicin; tob, tobramycin; ery, erythromycin; cli, clindamycin. †All isolates were negative for Panton-Valentine leukocidin except cluster 3.

**Figure:**
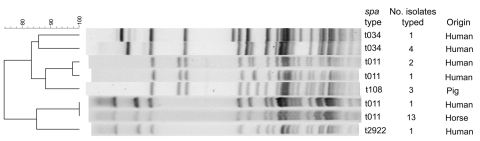
Dendrogram of *Apa*I–pulsed-field gel electrophoresis of methicillin-resistant *Staphylococcus aureus* clonal complex 398. Scale bar indicates percentage similiarity.

The human MRSA cases with CC398 isolates had no contact with each other. In 2 persons, the specimen was taken because of clinical symptoms, in 6 because of screening, and in 2 persons for unknown reasons (Table 2). Only the employee of the veterinary hospital had direct animal contact. For others, no direct contacts with horses or pigs were identified, and none owned a pet (Table 2).

**Table 2:** Human MRSA CC398 infections, Finland, January 2007–April 2009*

Patient age, y/sex	MRSA strain	Reason for taking specimen	Sample source	Animal contact	Other information
27/M	t011	Screening	Nares	Direct: horses	Staff member of veterinary hospital, direct link to horse epidemic
63/M	t011	Clinical symptoms	Wound secretion	None	Dentist, history of skin grafts, healthcare worker in family, travel history to the Canary Islands
82/F	t011	Clinical symptoms	Wound secretion	None	Several previous hospitalizations, multiple diseases including basal cell carcinoma; son has a dog
65/F	t011	Screening	Nares	Limited, visit to a horse farm (but not to the stable) 2 mo before MRSA isolation	Retired nurse, hospitalizations in Finland and in Switzerland due to trauma
57/M	t2922	Screening	Skin	None	Atopic skin, hospitalizations within previous year
82/M	t034	Unknown	Wound secretion	Limited, not specified	Several previous hospitalizations
78/M	t034	Unknown	Skin	Limited, occasional visits by a dog, had parakeets 20 y previously	Several previous hospitalizations
96/M	t034	Screening	Nares	None	Several previous hospitalizations
39/M	t034	Screening	Nares	None	Several previous hospitalizations, homeless, alcoholic, contact sport activities
42/M	t034	Screening	Nares	Unknown	Unknown

*MRSA, methicillin-resistant *Staphylococcus aureus*; CC398, clonal complex 398.

## Conclusions

Our nationwide population-based surveillance for MRSA showed the emergence of MRSA CC398 in 2007, and in total 10 findings in humans through April 2009. Molecular typing of both human and animal MRSA CC398 isolates identified 4 clusters; in only 1 was human carriage linked to occupation and related to the horse epidemic. Another cluster consisted of pig isolates, and 2 other clusters consisted of human isolates.

Animal contact is an established risk factor for MRSA CC398 (*4**,**5**,**12*). We identified direct animal contact for only 1 person. The other patients with MRSA lacked or had negligible animal contacts; instead, most of them had previous healthcare contacts. According to the national guidelines for MRSA control, screening is indicated when a patient has previously carried MRSA, has been exposed to a MRSA carrier, has been recently cared for at a facility where MRSA is endemic, or during an outbreak. Although some persons were possibly screened because of recent exposure to another MRSA carrier, direct transmission in these instances was unlikely because these persons carried different strains. Connections to Asia have previously been reported for persons with PVL-positive, t034 MRSA (*4*). Two cases of PVL-positive, *spa* type t034, MRSA infections in persons who had no contact with animals have also been reported from Sweden (*13*).

The proportion of CC398 of all MRSA in humans was far lower in Finland (0.09%) than in the Netherlands (30% in 2007) (*14*). Notably, the prevalence of MRSA in pigs is 40% in the Netherlands (*1*), whereas in Finland it is expected to be lower (*15*). The densities for pig and humans in the Netherlands are ≈60 and 30 times higher than those in Finland (http://epp.eurostat.ec.europa and www.stat.fi) respectively, and there are differences in screening policies. In the Netherlands, people in close contact with pigs or cattle are screened at hospital admission. It remains to be investigated whether the low occurrence of CC398 and lack of direct animal contacts among CC398 case-patients are related to lower occurrence of MRSA in animals, sparse pig and human populations, or screening policies in Finland.

Our questionnaire focused on healthcare-related risk factors and animal contacts. Although questions about traveling abroad were included, these data were difficult to interpret in relation to MRSA acquisition because travel is common. Furthermore, data for MRSA risk factors were not uniformly available for all patients. Another limitation was the lack of representative data for livestock, including pigs.

Except for the isolates from the horse epidemic, human and animal isolates were distinguished from each other by using a combination of molecular typing techniques. Additional typing by the *Apa*I–PFGE method may help in resolving outbreaks. Despite closely related and typical livestock associated *spa* types, *Apa*I PFGE distinguished the t108 pig isolates from the t011 human isolates at a possibly related level (4–5 band differences). In addition, the presence of 2 different SCC*mec* types among t011 and nonexistent links in time and space between human cases may suggest unrecognized transmission chains in the community.

## Supplementary Material

Technical Appendix Open reading frames of African swine fever virus.
