# Supplementary material for: Human Cases of Methicillin-Resistant Staphylococcus aureus CC398, Finland
Source: Emerg Infect Dis. 2010 Oct;16(10):1626–9. doi: 10.3201/eid1610.091571 (PMC3294383; doi:10.3201/eid1610.091571)
Supplement: Technical Appendix — Open reading frames of African swine fever virus. [file 09-1571-Techapp_4p.pdf]

Article DOI: 10.3201/eid1610.091571

Human Cases of Methicillin-Resistant *Staphylococcus aureus* CC398, Finland

Technical Appendix

Questionnaire for persons with an isolate of methicillin-resistant *Staphylococcus aureus* clonal complex 398

1. Indication of obtaining specimen

Clinical symptoms (yes/no)

Screening (yes/no)

Other (specify)

2. MRSA infection

Asymptomatic carriage (yes/no)

If no, type of MRSA infection:

Skin infections

Impetigo (yes/no)

Abscess (yes/no)

Erysipelas (yes/no)

Nail bed infection (yes/no)

Wound infection (yes/no)

Other skin infection (specify)

Other infections

Bursitis or arthritis (yes/no)

Eye infection (yes/no)

Ear infection (yes/no)

Other (specify)

3. Treatment of MRSA infection

Treatment (yes/no)

If yes:

Antimicrobials (specify)

Surgical treatment (yes/no)

Other treatment (specify)

4. Place of MRSA treatment

Acute-care hospital (yes/no)

Long-term care facility (yes/no)

Healthcare center/out-patient clinic (yes/no)

Other (specify)

5. Underlying conditions

Skin disease (specify)

Other underlying diseases (specify)

6. Household and other close contacts

Number of family members (number)

Person positive for CC398 is an immigrant (yes/no)

An immigrant in the household (yes/no)

A known MRSA infection or carriage in a close contact (such as a family member) (yes/no)

7. Preceding healthcare contacts

Acute-care hospital (yes/no, if yes where, how many times, and length of stay)

Long-term care facility (yes/no, if yes where, how many times, and length of stay)

Healthcare center/out-patient clinic (yes/no, if yes where, how many times)

Person positive for CC398 is a healthcare worker (yes/no)

A family member is a healthcare worker (yes/no)

Other (specify)

8. Occupation

The person positive for CC398 is working (yes/no, specify occupation)

Student (yes/no)

Conscript (yes/no)

Other (specify)

9. Hobbies

Contact sports (yes/no, if yes, specify)

10. Travels

Preceding travel outside Finland (yes/no)

11. Animal contacts

Has the person positive for CC398 been in contact with production animals (cattle, pig, sheep, chicken) or horses during recent years by visiting to farms, stables, or fairs? (yes/no)

If yes, do these animals have contacts to veterinary hospitals/clinics? (yes/no)

Has the person positive for CC398 been in contact with pets during recent years? (yes/no)

If yes:

Does he/she/family member own a pet (cat, dog, rodent, lizard, turtle, bird)? (yes/no)

Has he/she/family member a contact to a family with a pet? (yes/no)

Does the pet have contacts to veterinary hospitals/clinics? (yes/no)
